# Synergistic Anti-tumour Effects of Quercetin and Oncolytic Adenovirus expressing TRAIL in Human Hepatocellular Carcinoma

**DOI:** 10.1038/s41598-018-20213-7

**Published:** 2018-02-01

**Authors:** Hai Zou, Yong-fa Zheng, Wei Ge, Shi-bing Wang, Xiao-zhou Mou

**Affiliations:** 1 Clinical Research Institute, Zhejiang Provincial People’s Hospital, Hangzhou, 310014 China; 2 Key Laboratory of Cancer Molecular Diagnosis and Individualized Therapy of Zhejiang Province, Zhejiang Provincial People’s Hospital, Hangzhou, 310014 China; 3 Department of Oncology, Renmin Hospital of Wuhan University, Wuhan, 430060 China; 4 People’s Hospital of Hangzhou Medical College, Hangzhou, 310014 Zhejiang Province China

## Abstract

The combination of oncolytic adenoviruses and specific chemotherapy agents is fast emerging as a novel therapeutic approach for resistan the patocellular carcinoma (HCC) cells. A detailed analysis of the network between adenovirus and chemotherapeutic agents can help design an effective strategy to combat HCC. We sought to investigate whether a combined treatment of ZD55-TRAIL and quercetin can have an enhanced cell-killing effect on HCC cells. *In-vitro* experiments showed that quercetin can enhance ZD55-TRAIL mediated growth inhibition and apoptosis in HCC cells. In addition, we showed that quercetin reduced ZD55-TRAIL mediated NF-κB activation and down-regulated its downstream targets, which in turn promoted the pro-apoptotic action of ZD55-TRAIL. Furthermore, *in-vivo* experiments in mice injected with HuH-7 cells resulted in significantly greater reduction in tumour growth and volume following combined ZD55-TRAIL and quercetin treatment. In conclusion, we demonstrated that quercetin could sensitize human HCC cells to apoptosis via ZD55-TRAIL *in-vitro* and *in-vivo* and presented ZD55-TRAIL and quercetin combination as a suitable anti-HCC therapy.

## Introduction

Adenovirus (Ad), previously known as adenoidal-pharyngeal-conjunctival virus, was discovered in 1956 by Wallace Rowe *et al*. After observing its cytolytic effects on tumour tissue culture, the therapeutic efficacy of Ad was evaluated in cervical cancer cellsin 1956. Subsequently, the oncolytic properties of Ad have been exploited to improve anti-tumour therapy^1,2^ with the focus largely on improving and modifying certain aspects of Ad behavior in cancer cells, such as targeted infection, cancer cell specific replication, intra-tumour transduction and tumour immunogenicity, along-with generation of hybrid viruses with transgenes^3^. Hybrid oncolytic viruses armed with cytotoxic transgenes offers a potent anti-cancer therapeutic strategy. Certain properties of Ad, such as high titers, high transduction efficiency in both proliferating and quiescent cells and the ability to incorporate large transgenes, make it especially suitable for clinical viro-therapy. The most important feature of Ad vis-à-vis cancer therapy is the absence of integration into the host genome which obviates any virus induced mutagenesis in oncogenes or tumour suppressor genes. Therefore, compared to other oncolytic viruses like retrovirus, lentivirus and adeno-associated virus, Ad offers increased safety and potency^4–6^.

As many as 12 different oncolytic viruses, targeted against different cancer cells, are currently undergoing phase IIII clinical trials^7^. The H101Ad strain with the *E1B55K* gene deletion has been approved for treating head and neck cancerin China (Oncorine, Shanghai Sunway Biotech). In a previous study, we established a clinical gene therapy model using a novel recombinant Ad strain ZD55 with *E1B55K* deletion and Tumour Necrosis Factor-Related Apoptosis Inducing Ligand (TRAIL)^8^ insertion. The ZD55-TRAIL construct is a particularly attractive candidate for targeted tumour therapy since TRAIL-mediated apoptosis targets only tumour cells and not the adjacent healthy cells. Paradoxically, TRAIL can also exert an anti-apoptotic effect via nuclear factor NF-κB by the induction of the transcription of several anti-apoptosis sequences. This may be the possible underlying mechanism of the anti-TRAIL resistance seen in various cancer cells, although further studies are required to validate this hypothesis^9^. To circumvent the resistance to TRAIL, treatment strategies combining TRAIL with anti-cancer drugs have been designed where a synergistic increase in tumour cell apoptosis is seen that can be attributed to the activation of pro-apoptotic and the de-activation of pro-survival genes^10–13^.

Quercetin (with the IUPAC chemical name, 3,3′,4′,5,7-pentahydroxyflavone) is a class of flavonoid compounds with the hydroxyl-flavone backbone, found in onions, apple skin, lettuce, cauliflower, chilli peppers, celery and unsweetened cocoa. Interestingly, quercetin is a potential anti-cancer agent owing to its pro-apoptotic, anti-proliferative, anti-angiogenic and anti-inflammatory properties^14^. It exerts an antiviral activity in some hepatitis C patients^15^ and pro-apoptotic and anti-proliferative actions in numerous cancer cells including those of the breast, colon, lung, ovary and prostate.

In this study, we explored whether quercetin and ZD55-TRAIL hold a synergistic molecular effect against hepatocellular carcinoma (HCC). Our study demonstrates the synergistic anti-tumour activity of ZD55 TRAIL and quercetin for the first time. This combinatorial therapy killed HCC cells both *in vitro* and *in vivo*. Besides, quercetin also inhibitedthe (NF)κB mechanisms of action activated by ZD55 TRAIL which correlated with an enhanced degree of apoptosis. Our study presents the combination of ZD55 TRAIL and quercetin as a potent/revolutionary strategy for HCC therapy.

## Methodology

### Cell lines and viruses

The *Homo sapiens* HCC cell lines SMMC-7721, HepG2 and HuH-7 were sourced from the Cell Bank of Type Culture Collection of Chinese Academy of Sciences (CBTCCAS, Shanghai, China). All cell lines were cultured within the Dulbecco’s Modified Eagle’s Medium (GIBCO, Carlsbad, CA) mixed with 10% heat inactivated fetal bovine serum at 37 °C under 5-percent of CO_2_. The generation of the recombinant ZD55-TRAIL adenovirus has been previously described^16^ and the viruses were amplified in HEK293 cells. The Chinese Academy of Sciences provided grants for cell line use and the Ethics Committee of Zhejiang Provincial People’s Hospital (Hangzhou, China) approved the study and helped us by preparing the guidelines.

### *In vitro* cytotoxicity assay

Viability of the cells was evaluated by MTT (3-(4, 5-dimethylthiazol-2-yl)-2, 5-diphenyltetrazolium bromide) assay. Briefly, SMMC-7721, HepG2 and HuH-7 Cells were plated in 96 wells with the density of 1 × 104 cells/100 μl/well. The wells were incubated with varying concentrations of ZD55-TRAIL, quercetin or both for 48 hours. MTT (5 g/L) was added to the cells at 10 μl per well and incubated for another 4 hours. A DNA microplate reader was then used to measure absorbance at 570 nm.

### Hoechst 33342 staining

The cell were stained with Hoechst 33342 to detect apoptosis. HuH-7 cells were cultured at the growth medium immersed by only ZD55-TRAIL, only quercetin or the combination of both agents for 72 h and then incubated further for 30 min with Hochest 33342 (1 mg/ml) at 5 µl per well. The fluorescent cells can then be observed by the use of an inverted fluorescence microscope. Untreated cells were used as negative control.

### Flow Cytometric Analysis

HuH-7 cells were treated with ZD55-TRAIL (2MOI), quercetin (10 μM), or ZD55-TRAIL (2MOI) plus quercetin (10 μM) for 48 h. Apoptosis was in dicated by the V-FITC and PI double staining method as per manufacturer’s instructions. PI staining was used to determine cell cycle status of the cells. The stained cells were analyzed using FACS (FACS tarcytofluorometer, BD Biosciences).

### Western blot analysis

HuH-7 cells were pelleted, then bath (at least 2 times) with the phosphate-buffered saline. The lysis process can be induced by the application of RIPA buffer. Proteins were separated in a 12% SDS polyacrylamidegel and the gels were transferred onto PVDF (Polyvinylidene Fluoride) membrane. Specific proteins were detected by incubating the membranes with primary antibodies (dilutions in brackets) against the following: Adenovirus-5 E1A (1:1000), TRAIL (1:1000), capase-9 (1:1000), caspase-3 (1:1000), PARP (1:1000), GAPDH (1:1000), p65 (1:500), p50 (1:500), IκBα (1:500), Bcl-2 (1:500), FLIP (1:500), Bid (1:500) and Bax (1:500). The following secondary antibodies were used: anti-mouse (1:5000) and anti-rabbit (1:5000). Adenovirus-5 E1A, Capase-9, Caspase-3, PARP and GAPDH primary antibodies were bought from Santa Cruz Biotechnology and those against p65, p60 and IκBα from Cell Signalling.

### Animal experiments

The animal experimentation policies (Committee of Institutional Animal Care and Use, as well as our institutional’s) were followed carefully. Five weeks old male BALB/C nude mice were bought from the Shanghai Experimental Animal Center of Chinese Academy of Science and were adapted to our animal facility housing. HuH-cells were injected to the right flank of the mice and checked thrice weekly for any tumour development. Once the tumours grew to approximately 100–150 mm3, the mice were randomly grouped (6 mice per group) ZD55-TRAIL, quercetin, ZD55-TRAIL+quercetinand PBS as per treatment. ZD55-TRAIL was injected intra-tumourallyat 1 × 109 plaque-forming units (PFUs) per mouse while quercetin was injected intra-gastricallyat 150 mg/kg body weight. The control group was injected with 100 μl PBS continuously three times on alternate days. The dimensional indicators for tumour growth were measured every 3 days using vernier callipers and tumour volume (mm3) was calculated as (A × B2)/2, where A and B are the tumour length and width respectively.

### Statistical Analysis

Statistical significance was calculated using ANOVA analysis with Graph Pad 6.0. Data are presented in the form (mean ± standard deviation) and only when p < 0.05, the recorded values are regarded as significant.

## Results

### Quercetin enhances the anti-proliferative effect of ZD55-TRAIL in HCC cells

**Figure 1:**
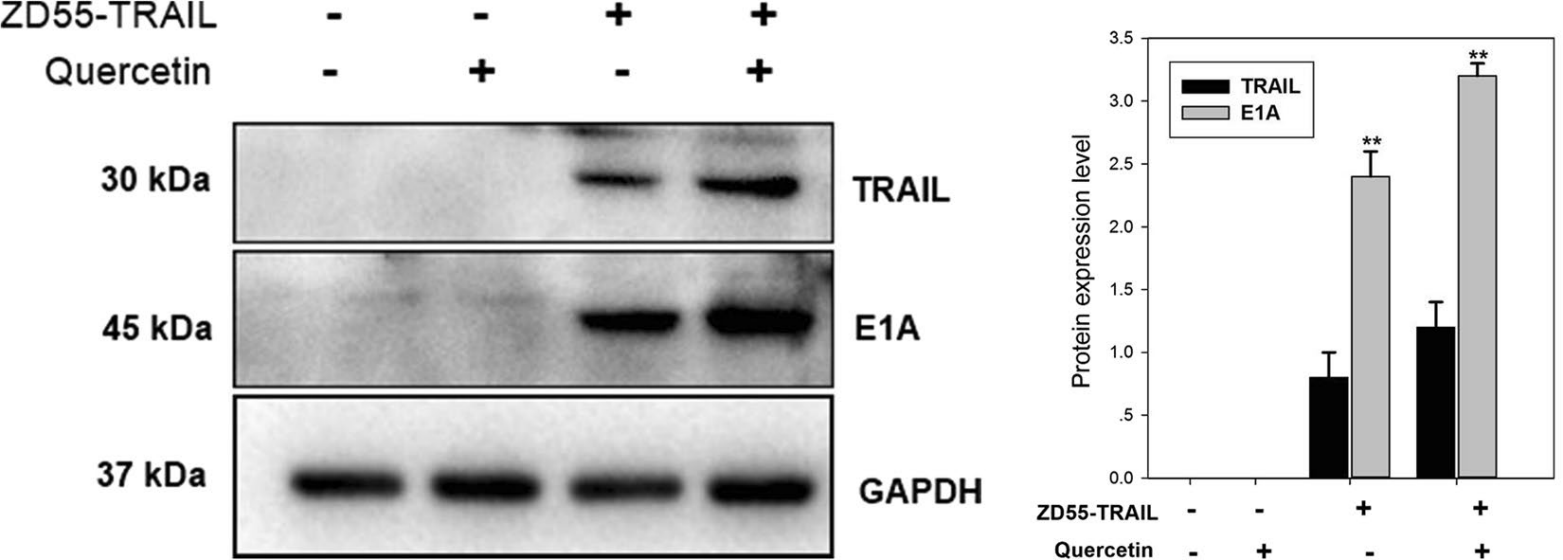
Characterization of ZD55-TRAIL. Notes: Identification of ZD55-TRAIL by Western blotting. HuH-7 cells were treated with Quercetin (10 μM), ZD55-TRAIL (2MOI) or Quercetin (10 μM) plus ZD55-TRAIL (2MOI); as a negative control, mock infected cells were included. Cells were lysed after 48 hours to analyze the expression of TRAIL and E1A proteins and GAPDH was used as the loading control. Semi-quantitative analysis of the differences in protein expression levels was performed by densitometry and expressed as percentages. Data are presented as mean ± SD and are representative of three separate experiments. (**Represents P < 0.01).

The ZD55 strain is a modified version of Ad 5 made by deleting the 55-kDa*E1B* gene, which enables it to replicate in various tumour cells. We packaged the TRAIL gene into the ZD55 virus to generate are combinant oncolytic Ad that we named ZD55-TRAIL (Fig. 1A). To validate the model, we confirmed the expression of the Ad E1A protein and TRAIL using Western blotting. HuH-7 cells infected with ZD55-TRAIL strongly expressed both E1A and TRAIL indicatinga high replicative capacity of ZD55-TRAIL in HCC cells (Fig. 1B). Similar results were obtained with ZD55-TRAIL infected HuH-7 cells treated additionally with quercetin.

**Figure 2:**
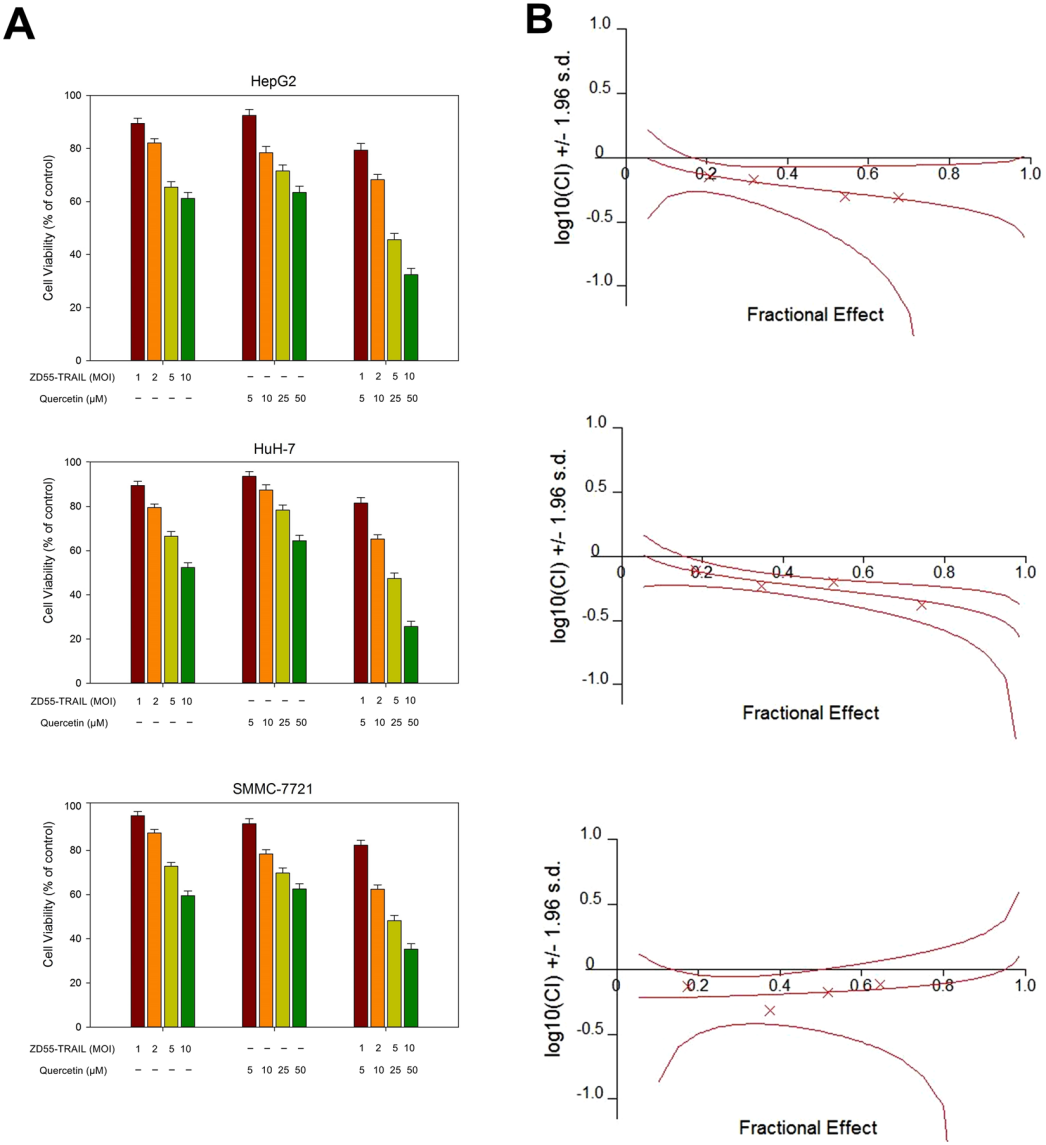
Quercetin enhances ZD55-TRAIL-mediated growth inhibition in HCC cells. Notes: (**A**) HCC cell lines HepG2, HuH-7 and SMMC-7721 were treated with ZD55-TRAIL (1, 2, 5, 10 MOI), quercetin (5, 10, 25, 50 μM), or both for 48 h and tested for viability by the MTT assay. The plots are representative of three independent experiments. (**B**) Synergistic effect of ZD55-TRAIL and quercetin on HCC cells was quantified by combination index (CIN) analysis and expressed as log_10_ (CIN) versus fraction affected. Confidence levels of 95% are shown wherever applicable. Synergism was analyzed by Chou–Talalay Combination Index (CI) using CalcuSyn software (Biosoft, Cambridge Place, Cambrige, UK). The simulated CI values, as shown by the middle curve, is expressed as the log_10_ (CI) ± 1.96 SD. Log_10_ (CI) > 0 indicates antagonism, Log_10_ (CI) = 0 indicates cumulative efficiency and Log_10_ (CI) < 0 indicates synergism.

The SMMC-7721, HepG2 and HuH-7 cell lines infected with ZD55-TRAIL displayed a higher incidence of cell death when treated with quercetin as per MTT assay (Fig. 2). These results clearly suggest an enhanced tumour killing effect of the combination of ZD55-TRAIL and quercetin.

### Quercetin augments ZD55 TRAIL mediated apoptosis in HCC cells

**Figure 3:**
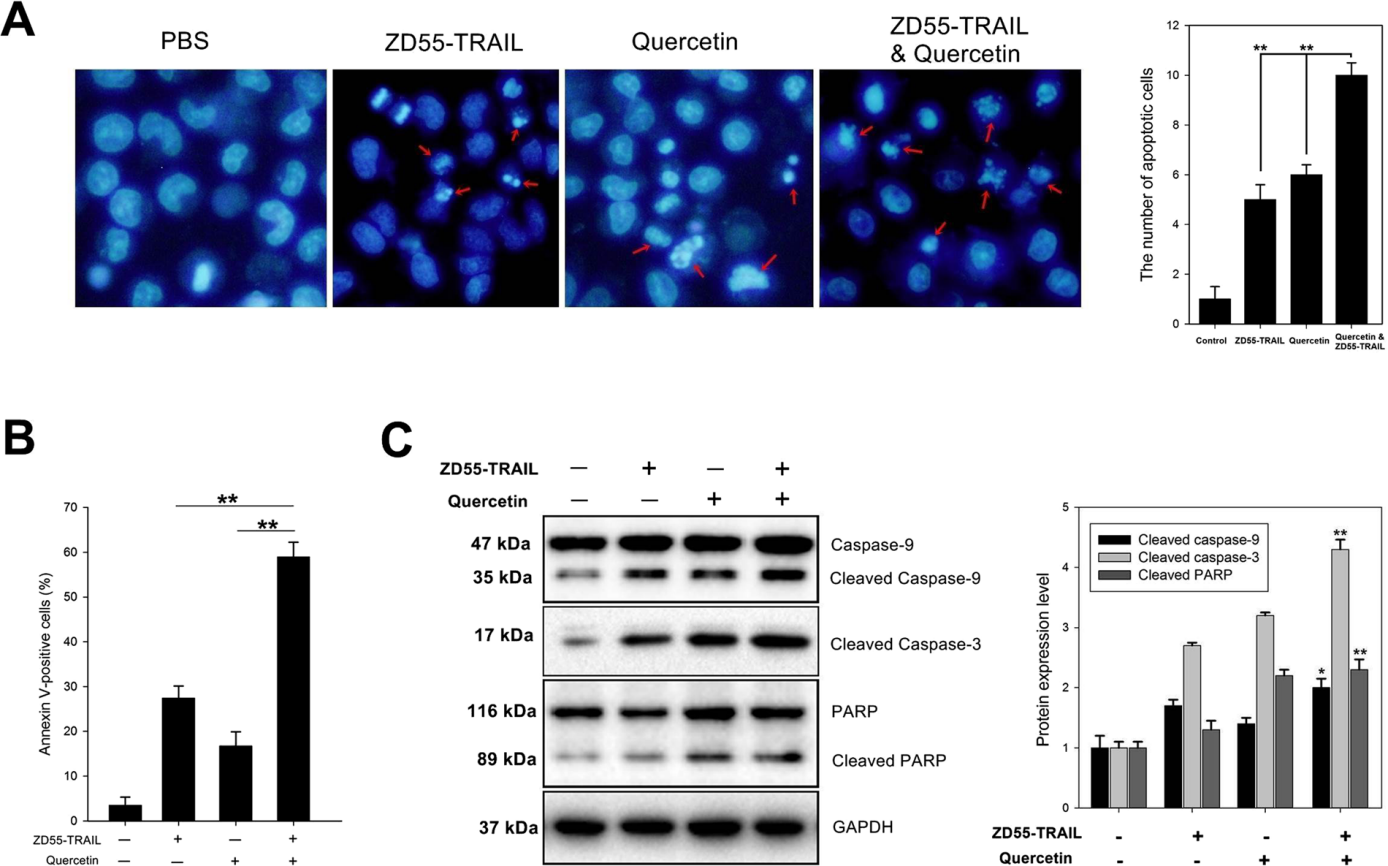
Quercetin enhances ZD55-TRAIL induced apoptosis in HCC cells. Notes: (**A**) Hoechst 33342 was used to detect apoptotic cells. HuH-7 were cultured with ZD55-TRAIL (2MOI), quercetin (10 μM), or ZD55-TRAIL (2MOI) plus quercetin (10 μM) for 72 hours and then treated for an additional 30 min with Hoechst 33342 (1 mg/ml; 5 µl per well). Fluorescent cells observed under the inverted fluorescence microscope. Apoptotic cells are indicated by the red arrows. Original magnification, ×400. Data are presented as means ± SD and are representative of three separate experiments, **P < 0.01. (**B)** ZD55-TRAIL (2MOI), quercetin (10 μM), or ZD55-TRAIL (2MOI) plus quercetin (10 μM) were used to treat HuH-7 cells, with uninfected cells as control. After 48 hours of incubation, apoptosis was determined by flow cytometry. Data are presented as means ± SD and are representative of three separate experiments, **P < 0.01. (**C)** HuH-7 cells were cultured with ZD55-TRAIL (2MOI), quercetin (10 μM), or ZD55-TRAIL (2MOI) plus quercetin (10 μM) for 48 h. Whole cell lysates were prepared and immunoblotted to detect proteins of anactivated caspase pathway, with GAPDH as the loading control. Differences in protein levels was determined by densitometry and expressed as percentages. Data are presented as mean ± SD and are representative of three separate experiments. (*Represents P < 0.05 and **represents P < 0.01, Combined treatment VS. ZD55-TRAIL-treatment).

HuH-7 cells treated with ZD55 TRAIL plus quercetin, ZD55 TRAIL or quercetin alone were stained with Hoechst 33342 to identify apoptotic cells. As shown in Fig. 3A, compared to ZD55 TRAIL treatment alone, combinatoric treatment with quercetin brought about the significantly increased apoptosis as illustrated by apoptotic bodies, degree of nuclear fragmentation and chromatin condensation. Apoptosis was assessed quantitatively in the ZD55 TRAIL infected cells using VFITC and PI (Fig. 3B). The rate of apoptosisin HuH-7 cells cotreated with quercetin was almost twice as high (58.9%) as that of cells not treated with quercetin (27.4%).

ZD55 TRAIL can also activate the caspase pathway as shown by the upregulation of caspase 9, caspase-3 and cleaved PARP by Western blotting. This pro-apoptotic effect was augmented by quercetin co-treatment (Fig. 3C).

### Quercetin inhibits ZD55-TRAIL-induced activation of NF-κB in HCC cells

**Figure 4:**
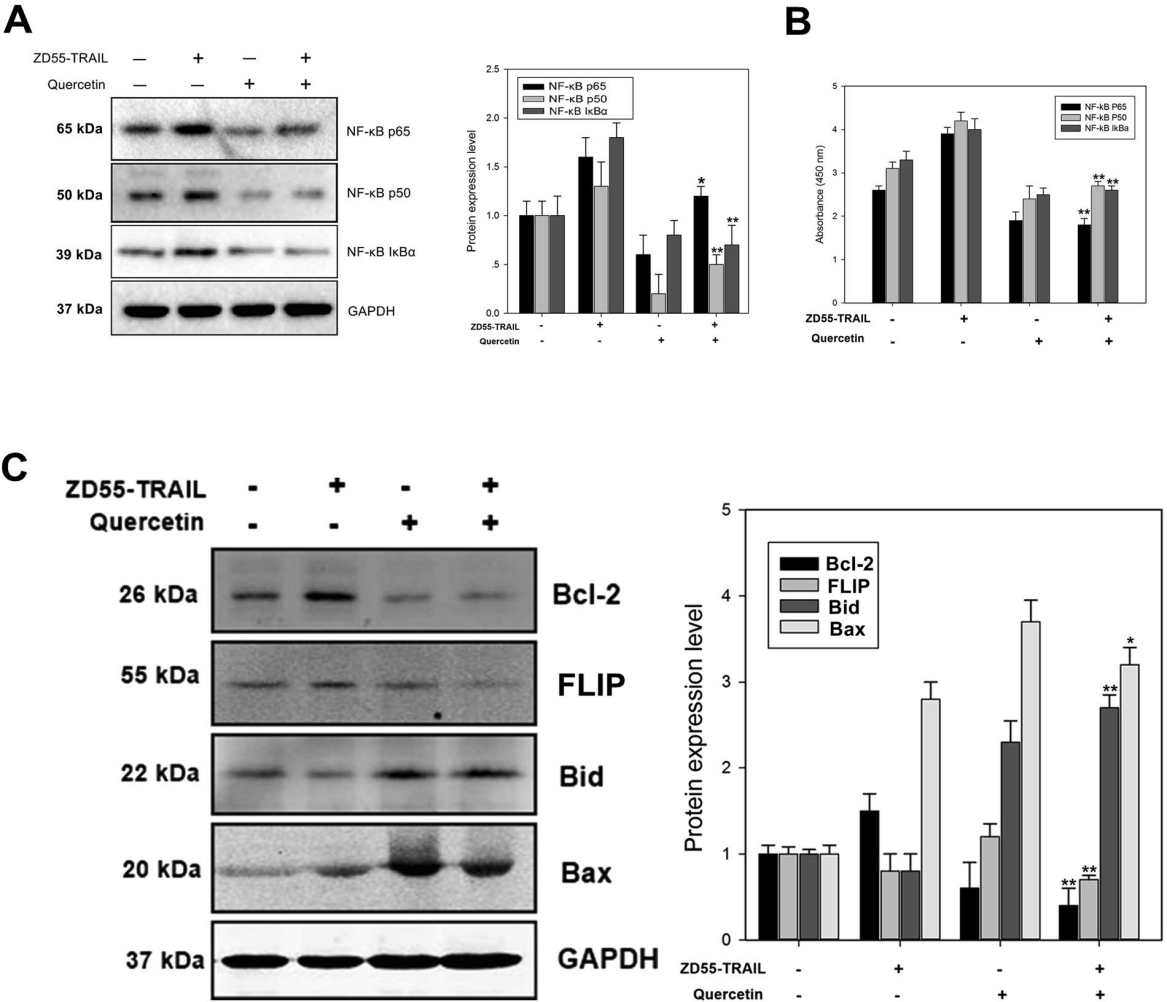
Quercetin inhibits ZD55-TRAIL induced NF-κB activation in HCC cells. Notes: HuH-7 cells were cultured with ZD55-TRAIL (2MOI), quercetin (10 μM), or ZD55-TRAIL (2MOI) plus quercetin (10 μM) for 48 h. Cell lysates were prepared and (**A**) Western blottingor (**B**) ELISA was performed to examine the changes in IκBα, p65, and p50 expression. GAPDH was used as the loading control. (**C**) Apoptosis-related proteins Bcl-2, FLIP, Bid and Bax were detected by Western blotting after treatment with ZD55-TRAIL (2MOI), quercetin (10 μM), or ZD55-TRAIL (2MOI) plus quercetin (10 μM) for 48 h. Differences in protein levels was determined by densitometry and expressed as percentages. Data are presented as mean ± SD and are representative of three separate experiments. (*Represents P < 0.05 and **represents P < 0.01, Combined treatment VS. ZD55-TRAIL-treatment).

To test the ability of quercetinin inhibiting NF-κB-dependent transcription and sensitizing HCC cells to ZD55-TRAIL mediated apoptosis, we determined the expression of the downstream targets of NF-kB, namely IκBα, p65, and p50, through Western blotting and ELISA. As shown in Fig. 4A, the expression of p65, p50 and IκBα was significantly more reduced upon quercetin and ZD55-TRAIL co-treatment than ZD55-TRAIL treatment alone, indicating that quercetin can downregulate ZD55-TRAIL-mediated NF-κB activation and the latter’s downstream transcriptional targets, thus enhancing ZD55-TRAIL Finduced apoptosis.

For additional mechanistic analysis of the synergistic effect of quercetin and ZD55-TRAIL, the expression of apoptosis-inducing family proteins like Bcl-2, FLIP, Bid and Bax were also analyzed by Western blotting. As shown in Fig. 4C, cells treated with quercetin and ZD55-TRAIL displayed lower levels of the anti-apoptotic Bcl-2 and FLIP, and higher levels of pro-apoptotic Bid and Bax compared to cells treated with only quercetin or only ZD55-TRAIL. These findings indicate that the molecular interaction of quercetin and ZD55-TRAIL increases apoptosis in pancreatic cells by regulating apoptosis-related determinants/messengers such as Bcl-2, FLIP, Bid and Bax.

### Quercetin enhances ZD55-TRAIL mediated inhibition of HCC tumour growth *in vivo*

**Figure 5:**
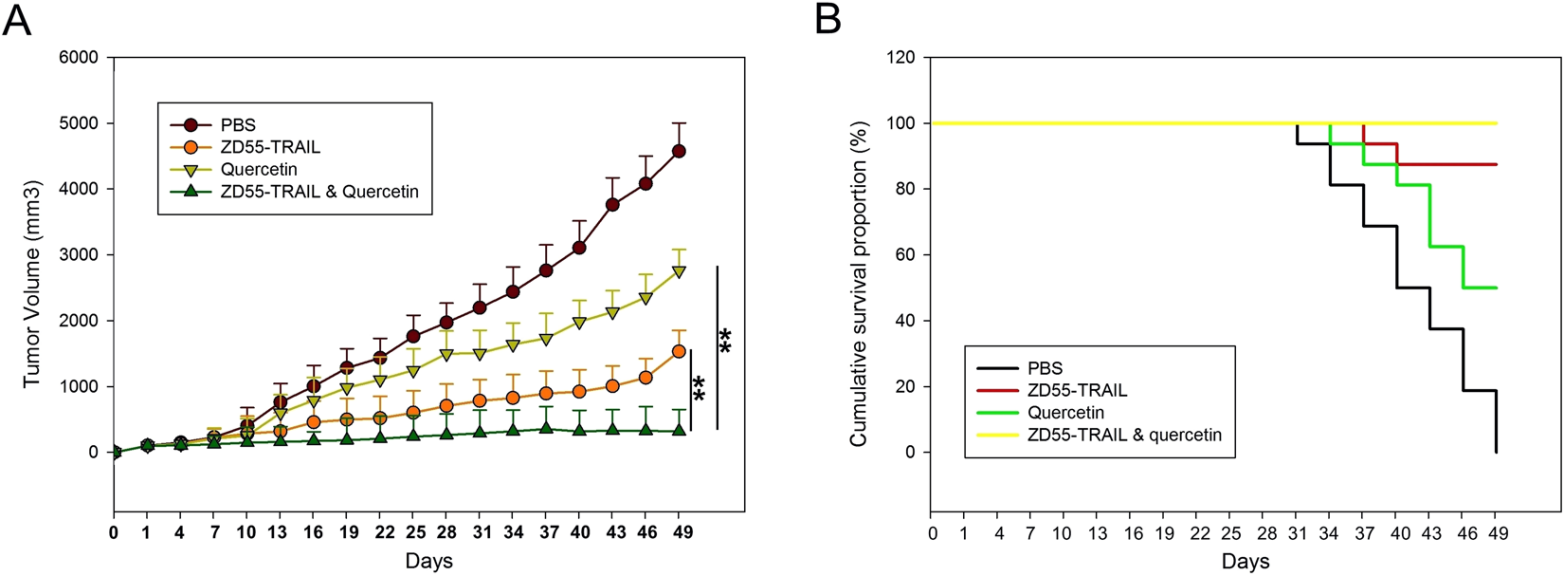
Synergistic effects of ZD55-TRAIL and quercetin *in vivo*. Notes: (**A**) Tumour volume was measured post-treatment at different time points. At the beginning of the drug injection, average tumour volumes in each group were 105 mm^3^ (PBS), 101 mm^3^ (ZD55-TRAIL), 112 mm^3^ (Quercetin) and 110 mm^3^ (ZD55-TRAIL & Quercetin). Data are presented as means ± SD (n = 6). **P < 0.01. (**B**) Pictures showing the differences in tumour dimensions of the three treatment groups on day 49 post-treatment when the mice were finally sacrificed.

To validate the *in vivo* therapeutic effects of quercetin and ZD55-TRAIL, a murine HCC tumour xenograft model was established by HuH-7 cells. Following HCC cell injection, the mice were observed for a period of 49 days and tumour growth was analyzed as described. As shown in Fig. 5A, the mean tumour volume was significantly decreased in animals that received intra-tumoural injections of quercetin, ZD55-TRAIL, or both, compared to those who received only PBS injections. In support of our hypothesis, ZD55-TRAIL and quercetin co-treatment was more effective in reducing tumour volume compared to quercetin (*P* = 0.001) and ZD55-TRAIL treatments alone (*P* = 0.002). In addition, the co-treated group also had a higher survival rate compared to the PBS, quercetin or ZD55-TRAIL groups (Fig. 5B).

## Discussion

Synergistic targeted therapy is the ideal treatment for cancer since tumours are made of genetically diverse clones which can easily become resistant to a single oncolytic agent^17,18^. The idea is to combine two or more agents, each with a different anti-tumour mechanism, to cumulatively increase anti-tumour activity without affecting healthy cells or causing systemic side effects. Oncolytic viro-therapy, although targeted specifically towards cancer cells, has not been effective so far as a solo anti-tumour therapy^19^. Clinical trials incorporating chemotherapy^20–22^ or radiation therapy^23^ in addition to viro-therapy show significantly enhanced, and synergistic, antitumour activity. In particular, cytotoxic chemotherapeutic agents increase the potency of oncolytic viruses. Therefore, the interactions between oncolytic adenovirus and chemotherapeutic agents need to be studied in greater detail to help design more efficacious therapeutic strategies to combat cancer^2,24^.

TRAIL, a member of the tumour cell-death factor super family, is secreted largely by normal cells but triggers apoptosis only in tumour cells on account of specific death receptors expressed on the latter. Despite being a promising oncolytic agent, the clinical application of TRAIL is fraught with certain major limitations as a result of emergence of resistant clones^25^. Recent studies have shown that merging TRAIL with other chemotherapeutic agents can cumulatively increase tumour cell apoptosis by activating pro-apoptotic and suppressing pro-survival gene expression^8,10^. TRAIL resistant clones can be sensitized to TRAIL induced apoptosis by adding potent anti-cancer drugs, antioxidants, targeted small molecules or irradiation in the treatment regimen^26,27^. We hypothesized that quercetin can augment TRAIL induced apoptosis through an alternate mechanism. Our results show that co-treatment with quercetin and ZD55-TRAIL can cumulatively lower HCC cell survival both *in vitro* and *in vivo* and markedly increase apoptosis by activating caspase-9, caspase-3 and PARP. Furthermore, quercetin also sensitized the TRAIL-resistant cancer cells to apoptosis.

Since quercetin is known to block the NF-κB signalling pathway and its downstream anti-apoptotic and metastatic factors^28,29^, we next hypothesized that this inhibition of NF-κB-dependent transcription is probably the underlying mechanism of quercetin mediated sensitization of TRAIL-resistant cells. We therefore analyzed the NF-κB-signalling pathway in HCC cells treated with quercetin and ZD55-TRAIL by looking at the expression of the individual elements of the pathway. Quercetin significantly reduced the expression of IκBα, p65, and p50, thereby inhibited NF-kB signalling. In conclusion, quercetin inhibits the ZD55-TRAIL mediated activation of NF-κB and its anti-apoptotic target genes, thus promoting ZD55-TRAIL induced apoptosis.

To summarize, we have demonstrated that quercetin sensitizes human HCC cells to ZD55 TRAIL induced apoptosis and have presented a novel targeted anti-HCC therapy that relies on a combination of quercetin and ZD55-TRAIL.

## Electronic supplementary material

Figure S1
