# Supplementary material for: Synergistic Anti-tumour Effects of Quercetin and Oncolytic Adenovirus expressing TRAIL in Human Hepatocellular Carcinoma
Source: Sci Rep. 2018 Feb 1;8:2182. doi: 10.1038/s41598-018-20213-7 (PMC5794998; doi:10.1038/s41598-018-20213-7)

Supplementary Information

Synergistic Anti-tumor Effects of Quercetin and Oncolytic Adenovirus expressing TRAIL in Human Hepatocellular Carcinoma

Hai Zou, Yong-fa Zheng, Wei Ge, Shi-bing Wang, Xiao-zhou Mou

Clinical Research Institute
Zhejiang Provincial People's Hospital, Hangzhou 310014, China

Supplementary Figure S1 Schematic structure of ZD55-TRAIL.

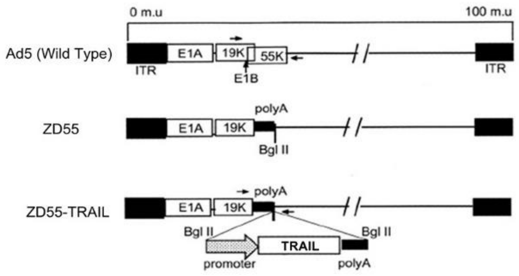

Notes: In ZD55- TRAIL, the E1B 55-kDa gene (bp2269-3327) was replaced by SV40 polyA and the expression box of TRAIL.

Supplementary Figure S2 Full-length gels and blots images.

Figure 1

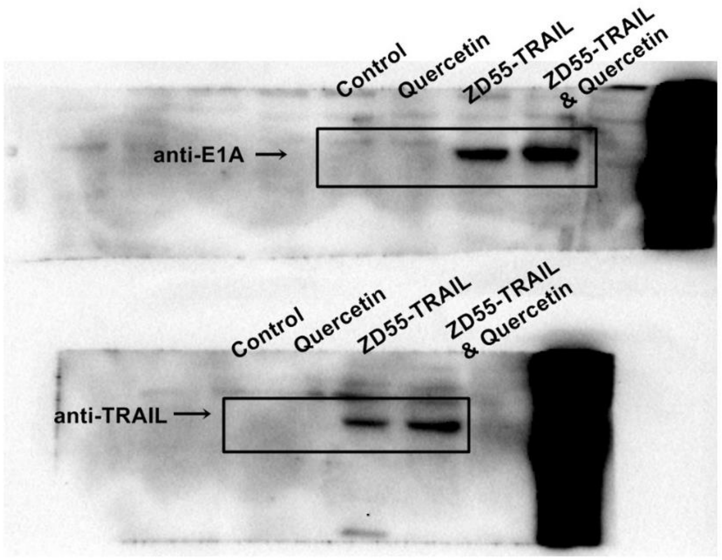

Figure 3C

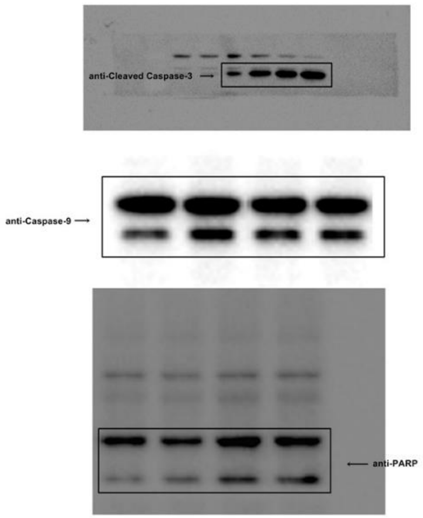

Figure 4

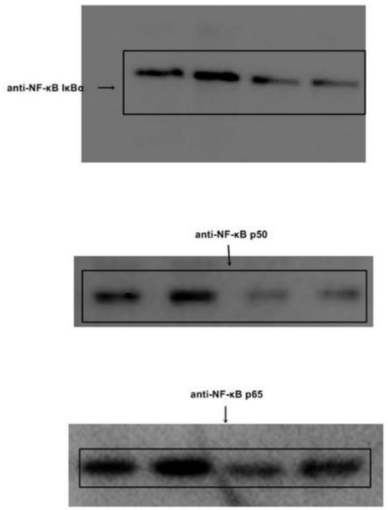

Supplement: Supplementary file 1 — Figure S1 [file 41598_2018_20213_MOESM1_ESM.pdf]
